# Reframing dopamine: A controlled controller at the limbic-motor interface

**DOI:** 10.1371/journal.pcbi.1011569

**Published:** 2023-10-17

**Authors:** Kevin Lloyd, Peter Dayan

**Affiliations:** 1 Max Planck Institute for Biological Cybernetics, Tübingen, Germany; 2 University of Tübingen, Tübingen, Germany; Scuola Internazionale Superiore di Studi Avanzati, ITALY

## Abstract

Pavlovian influences notoriously interfere with operant behaviour. Evidence suggests this interference sometimes coincides with the release of the neuromodulator dopamine in the nucleus accumbens. Suppressing such interference is one of the targets of cognitive control. Here, using the examples of active avoidance and omission behaviour, we examine the possibility that direct manipulation of the dopamine signal is an instrument of control itself. In particular, when instrumental and Pavlovian influences come into conflict, dopamine levels might be affected by the controlled deployment of a reframing mechanism that recasts the prospect of possible punishment as an opportunity to approach safety, and the prospect of future reward in terms of a possible loss of that reward. We operationalize this reframing mechanism and fit the resulting model to rodent behaviour from two paradigmatic experiments in which accumbens dopamine release was also measured. We show that in addition to matching animals’ behaviour, the model predicts dopamine transients that capture some key features of observed dopamine release at the time of discriminative cues, supporting the idea that modulation of this neuromodulator is amongst the repertoire of cognitive control strategies.

## Author summary

Evolution provides us with behavioural tendencies that are usually adaptive, but sometimes interfere with our goals. The glimpse of a cream bun in the bakery window may lure us to actions defeating our dieting aims; the sound of a loud car horn as we cross a street may cause us to freeze reflexively when it would be better to hasten out of the way of oncoming traffic. Such ‘Pavlovian’ influences over behaviour, in these examples respectively promoting active approach and behavioural inhibition, involve the action of neuromodulators, such as dopamine, in subcortical brain areas. Here, we consider the possibility that one strategy the brain employs to attempt to control such occasionally errant processes is to manipulate the neuromodulatory signal itself. We examine experimental results from two rodent studies that measured subcortical dopamine release while rats made active responses to evade punishment, or inhibited their responses to gain reward. We build a model of the rats’ behaviour that includes the possibility of controlling dopamine release, and show that the model can capture key patterns in the data. This lends support to the idea that the brain may sometimes exert control by manipulating neuromodulation itself.

## Introduction

Evolution has endowed animals with behavioural tendencies such as approaching and engaging with sources and predictors of food, and freezing or withdrawing from sources and predictors of punishment [1]. Such ‘Pavlovian’ inductive biases [2] provide an effective way to obviate the need for learning in situations that are common and occasionally critical [3], and exert a powerful influence on behaviour [4, 5]. However, they can also lead to counterproductive Pavlovian-instrumental conflict—‘Pavlovian misbehaviour’ [2]—if animals must act vigorously to avoid predicted punishment, or withhold actions to gain potential reward [2, 6–9]. They then need to be suppressed or supplanted, in what can be interpreted as a form of cognitive control.

One contributor to the Pavlovian-instrumental conflict may be the neuromodulator dopamine (DA), in a clash between its dual roles in positive reinforcement and motivational vigour [10–12]. Evidence from canonical versions of these conflict paradigms suggests that DA in the core of the nucleus accumbens (NAc), at least when performance is successful, follows its motivational, rather than its reinforcement, role, with enhanced DA concentrations being observed during active avoidance [13, 14] and suppressed DA concentrations when behavioural suppression is required to gain reward [15].

Partly inspired by the two-factor theory of active avoidance [16, 17], Boureau and Dayan [18] suggested that such DA dynamics might be conceptualized as arising from a shift of the origin in a valence-action space. In the active avoidance case, a shift to a negative valence corresponding to expected punishment means that a neutral outcome (avoidance) appears positive; enhanced release of DA associated with the prospect of safety could then play a role in energizing the necessary active avoidance response. Conversely, when behavioural suppression is required to gain reward, a shift of the origin to the associated positive valence means that a neutral outcome (no reward) appears negative; suppression of DA release associated with the prospect of this loss may promote behavioural inhibition.

Subsequent work [19, 20] elaborated on this suggestion in relation to active avoidance, but did not provide a process account of the reframing required to turn a situation that is, at best, neutral into one that appears positive. Furthermore, an account of the opposite reframing—to turn a situation that is, at worst, neutral into one that appears negative—has been lacking.

In the current work, we address these shortcomings via a modelling framework that characterizes the putative reframing operations of [18], and associated effects on DA signalling, as resulting from internal cognitive control actions. As modelling targets, we focus on two recent experimental studies in rodents, both involving measurement of NAc DA release: a study by Gentry et al. [21] involving active avoidance, and a study by Syed et al. [15] involving behavioural suppression to obtain reward. After briefly outlining the main idea of the model, we describe these experiments and their principal findings, and show how our model may account for certain critical features of the observed dynamics of DA release associated with cue and control (leaving out the outcome). We also consider the important issue of how the putative reframing mechanism could remain stable given the plasticity typically associated with DA release.

## Results & discussion

### Model

In instrumental or operant conditioning [1], animals learn to make responses given particular sensory stimuli. These responses are based, at least initially [22], on the outcomes that are contingent on those responses—animals typically prefer responses that lead to greater rewards or lesser punishments, and avoid responses that lead to greater punishments or lesser rewards. Conversely, in Pavlovian or classical conditioning, animals learn the predictive relationship between sensory stimuli and affectively important outcomes, and then those stimuli come to elicit a set of automatic, conditioned, responses irrespective of the contingency between those responses and the outcomes [1]. Pavlovian responses include approach and engagement for appetitive predictors, and withdrawal and inhibition for aversive predictors.

The involuntary nature of conditioned responses implies that difficulties can arise in situations such as active avoidance (in which animals avoid a punishment only if they act in a short time after a predictive cue) and omission schedules (in which animals will receive a reward following a cue only if they do *not* act). This is because the instrumental requirement (acting or withholding, respectively) is directly opposed by the Pavlovian conditioned response (freezing to the punishment predictor, or engaging to the reward predictor) [2, 23, 24].

In a simple case in which stimuli *s* are potentially associated with the emission (‘Go’) or withholding (‘NoGo’) of active responses, this was operationalized in [25] by state-action values (*Q*-values [26]) *Q*(*s*, Go) and *Q*(*s*, NoGo), which respectively capture the long-run benefit of responding or not, being additively corrupted by a quantity *ωV*(*s*) proportional to the predicted affective value of the state.

First consider the case of the omission schedule. In this case, provided that performance is at least somewhat successful, *s* will predict some reward, and so *V*(*s*) > 0 will be positive. Then, assuming that *ω* > 0, the Pavlovian factor will *boost* the propensity to act/‘Go’—and so may in some cases interfere with the very behaviour (suppression of action) that led to success in the first place.

Noting (i) that the TD error [27] typically associated with state *s*_*t*+1_,

$$\begin{array}{c} \delta_{t+1}^{}=r_{t}+V\left(s_{t+1}\right)-V\left(s_{t}\right), \end{array}$$ (1)

is just the *value* of that state, *V*(*s*_*t*+1_), if there is no extrinsic reward at that moment (*r*_*t*_ = 0) and no precise prior expectation (*V*(*s*_*t*_) = 0), and (ii) the dopaminergic realization of this TD error [10, 28–30], then one contributor to this Pavlovian effect might be the incentive salience-associated release of DA [31]; this would energize action [32], perhaps via its action on direct and indirect pathways in the striatum [12].

Conversely, in the case of active avoidance, if the animal is at least partially incompetent and so receives some shocks, *V*(*s*) < 0 will be negative. In this case, the Pavlovian factor, *ωV*(*s*), will *suppress* the propensity to act (i.e., favours ‘NoGo’), e.g., by promoting freezing or withdrawal. It is notably less clear that this arises only from below-baseline DA at *V*(*s*_*t*+1_)<0 as opposed, for instance, to the activity of a separate opponent system [18, 33] or a non-dopaminergic boosting of the indirect pathway in the striatum [12, 34].

Our central conceit is that the coupling of DA with action provides both the opportunity and need for a form of cognitive control in which DA release is manipulated by a *reframing* of values. This generalizes the suggestion [18, 20] that the origin of the valence axis of the affective circumplex can be adjusted. Such control might fully determine a particular trajectory for DA release; however, we explore a more limited construct in which it induces new, counterfactual [35], states associated with default expectations, with an effect on dopamine concentrations associated with the discriminative cues in these experiments. This induction then influences DA.

We provide the essence of the model in the results below; full modelling details can be found in the Methods.

### Active avoidance

**Fig 1:**
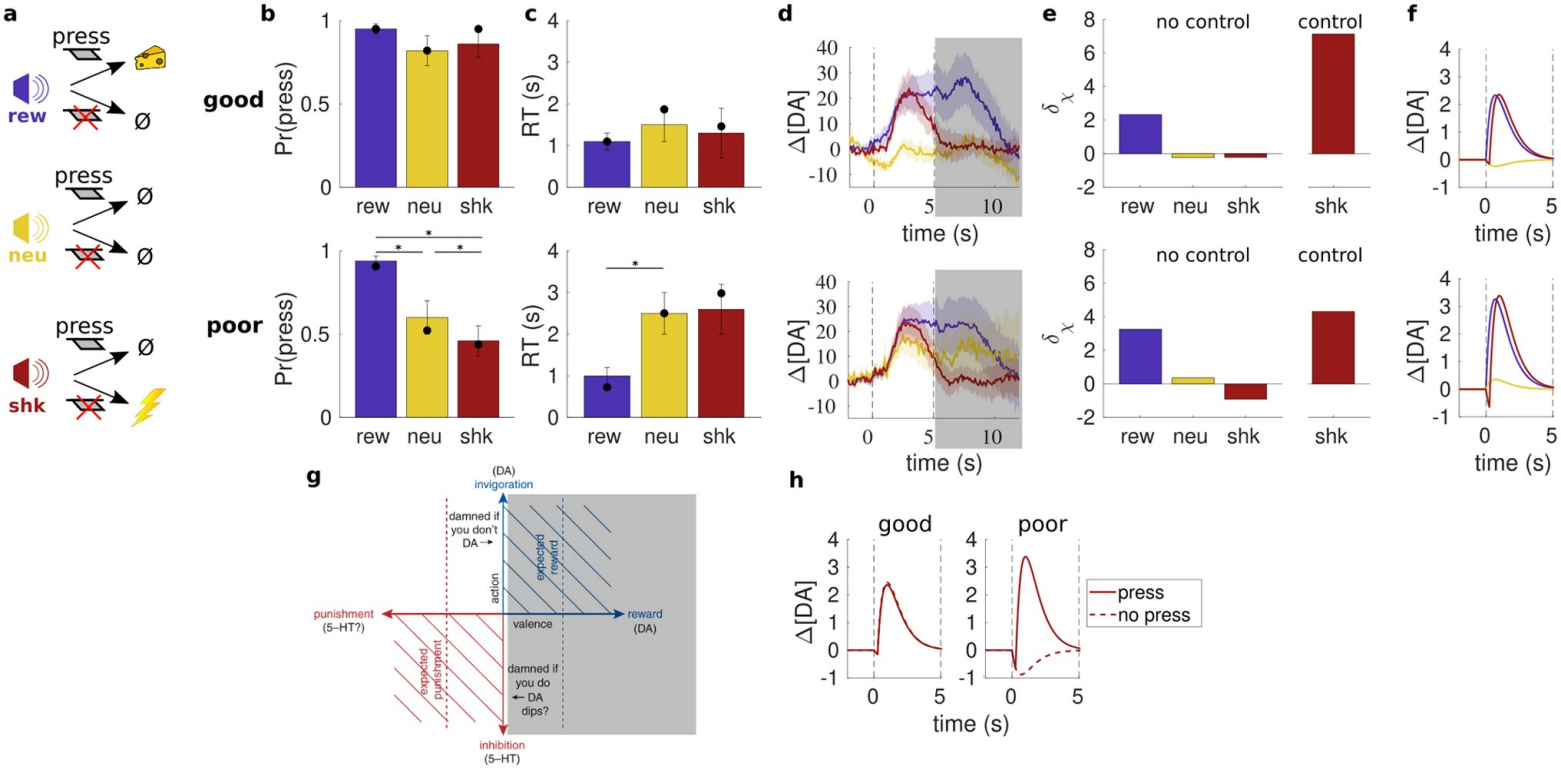
Mixed-valence task of Gentry et al. [14]. (**a**) On each trial, a tone indicated whether a lever press within 10 s would yield reward, have no consequence, or avoid a scheduled shock. (**b,c**) Good-avoiders (upper) pressed often and quickly; poor-avoiders (lower) pressed less often and more slowly on shock and neutral trials (*indicates significance, *p* < .05). Filled circles indicate model fit. (**d**) Average cue-aligned (nanomolar) NAc DA release (±SEM) on press trials for each trial type (vertical dashed lines indicate cue onset at 0 s and lever insertion at 5 s). Our focus is on DA release arising in response to the tone; the shaded region covers lever insertion and subsequent events. (**e**) Cue-evoked TD errors predicted by model (arbitrary units). (**f**) Average DA release predicted by model on press trials (arbitrarily scaled). (**g**) Putative shifting of origin leftwards in the affective circumplex to promote DA release and approach to safety in the active avoidance case (adapted from [18]). (**h**) Predicted DA release for press vs. no-press shock trials. (Figures b–d adapted from [14].)

A particular example involving active avoidance is provided by Gentry et al. [14], who used fast-scan cyclic voltammetry (FSCV) to examine DA release in the core of the NAc during performance of a mixed-valence task. In one class of trials, rats heard a tone telling them that they had to press a lever within a 10 s response window to avoid a shock (Fig 1a). Half the animals often struggled to respond actively in time (Fig 1b and 1c), and showed higher rates of freezing—a typical example of Pavlovian-instrumental conflict. However, across the population, on trials when they did press, cue-elicited DA release was similar just after shock or reward cues (Fig 1d), notably being stimulated rather than suppressed.

For convenience, we write *s*_pre_ for the state before the tone that indicates trial type, with *V*(*s*_pre_) ≃ 0 (from long, subjectively uncertain, inter-trial intervals), and *s*_*χ*_, *χ* ∈ {rew, neu, shk}, for the different states entered depending on the tone. Then, for shock trials and imperfect avoidance, *V*(*s*_shk_) < 0. Thus, specifying the terms in Eq (1), and noting that *r*_*t*_ = 0 at the time the stimulus is presented, we would have

$$\begin{array}{c} \delta_{\text{shk}}^{}=V\left(s_{\text{shk}}\right)-V\left(s_{\text{pre}}\right)<0, \end{array}$$ (2)

promoting Pavlovian inhibition; as positive feedback, this would make avoidance harder, thus making *V*(*s*_shk_) more negative and exacerbating the problem.

Our assumption is that for shock trials, the deployment of cognitive control instills a counterfactual state *s*_fail_ that substitutes for *s*_pre_, with *V*(*s*_fail_) ≪ 0 quantifying the full explicit cost of the shock. Then,

$$\begin{array}{c} \delta_{\text{shk}}^{}=V\left(s_{\text{shk}}\right)-V\left(s_{\text{fail}}\right)>0, \end{array}$$ (3)

promoting Pavlovian action. This relocation of the origin of the affective circumplex to the negative affective value associated with presumed failure and thus the shock (Fig 1g) harmonizes Pavlovian and instrumental control in the service of active responding—and would explain the positive (“damned if you don’t”) DA transient for successful avoidance in [14].

To test this, we fitted a model (see Methods) that incorporates Pavlovian influences, via an effective value of *ω*, and a probability of employing control to the animals’ behaviour (Fig 1b and 1c). Averaging over the resulting mixture of differential TD errors for no-control vs. control shock trials (Fig 1e) then indeed implies a net-positive DA signal on trials where animals successfully avoid shock (Fig 1f), assuming that the TD signal is conveyed by DA transients (noting that the modelled concentrations are arbitrarily scaled in the figure). The predicted suppression of DA release for poor avoiders on failed avoidance trials (Fig 1h) would be consistent with such failures of control, and with observations [36, 37] that enhanced or suppressed DA release given a warning cue predicts successful or failed active avoidance. Of course, given small *ω*, successful avoidance could be achieved without control.

We briefly note some discrepancies between the data and model behaviour. Firstly, the relative magnitude of DA release on poor-avoidance neutral trials in the model is lower than is observed in the data, a characteristic also noted in [20]. One possibility is that there is partial confounding of cues, leading to a degree of generalization in the DA response [38, 39]. By contrast, we assumed perfect knowledge of the relationship between cues and trial types. Secondly, the model appears to predict greater cue-evoked DA release (on average) during successful avoidance for poor avoidance sessions (cf. Fig 1h), something which is not evident in Fig 1d. One thing to note here, however, is that the plots in Fig 1d depend on splitting sessions according to a particular operationalization of good vs. poor avoidance (in [21], this was based on the relative performance in neutral and shock trials, rather than on performance in shock trials alone); such categorization may have obscured relationships that would be apparent by instead considering avoidance on a continuum. Indeed, an intriguing observation made by Gentry et al. [21] was of a significant negative correlation between the rate of successful avoidance and the magnitude of shock cue-evoked DA release on avoidance trials—i.e., the worse the avoidance performance, the larger the DA release in response to the shock cue on avoidance trials. Given the relative paucity of data, we were not able to fit individual animals/sessions, but we certainly find this correlation suggestive.

Finally, we only set out to model the DA transients associated with the cue (i.e., the 5 s period between cue onset and lever insertion, ending at the shaded region in Fig 1d). However, Fig 1d suggests that even within this limited time window, cue-evoked DA release on press trials is prolonged for the reward cue relative to the shock cue. As discussed in our previous work [20], one possibility is that this arises from incomplete ‘predicting away’ of the rewarding outcome on those trials, something which we have not addressed here. Indeed, relatively persistent DA release in response to cues and outcomes that are (in principle) perfectly predictable appears to be quite common in rodent experiments (e.g., [40–42]). We also note the intriguing hint of a second positive DA transient on poor avoidance neutral trials around 7–8 s that presumably coincides with lever retraction—suggesting that the lever may have acquired net negative valence.

### Go/No-Go

**Fig 2:**
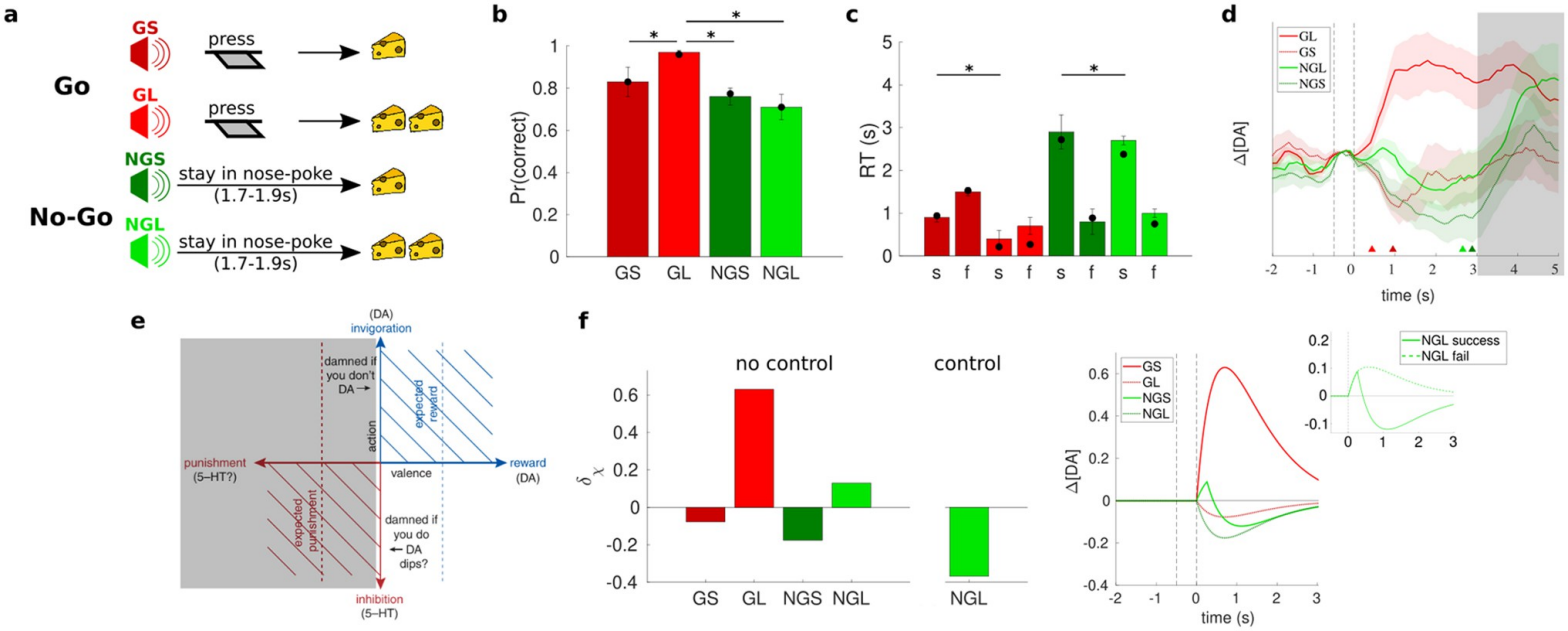
Go/No-Go task of Syed et al. [15]. (**a**) On each trial, a tone indicated whether the animal should leave the nose-poke and press a lever (‘Go’) to gain a small (GS) or large (GL) reward; or remain in the nose-poke until the tone turns off (‘No-Go’) to gain a small (NGS) or large (NGL) reward. On No-Go trials, the duration of the tone was randomly jittered between 1.7–1.9 s on each trial. (**b,c**) Average success rates and RTs (±SEM) for each trial type; the latter are split further into successful (s) and failed (f) trials (*indicates significance, *p* < .05). Filled circles indicate model fit. (**d**) Average cue-aligned change in DA (±SEM) for each trial type on successful trials; triangles indicate mean RTs. Again, our focus is on DA release associated with the cue, and in this case we simply focus on the first 3 s following cue onset (i.e., before the shaded region). (**e**) Putative shifting of origin rightwards in the affective circumplex to suppress action in light of predicted reward (adapted from [18]). (**f**) Cue-evoked TD errors predicted by model. (**g**) Average DA predicted by model on success trials. Inset: average DA predicted by model on successful vs. failed NGL trials. (Figures b–d adapted from [15].)

While Pavlovian influences may take the form of counterproductive behavioural inhibition in the case of active avoidance, they may also appear as unhelpful behavioural *activation* when suppression would be preferable. Syed et al. [15], used a Go/No-Go task in which one of four auditory cues indicated whether rats had to leave a nose-poke (‘Go’) and execute an active response (press a lever twice) or stay in the nose-poke until the tone turned off (‘No-Go’) in order to get a small or large reward (Fig 2a). Animals were reliably successful on Go large-reward (GL) trials, but less so on No-Go large-reward (NGL) trials (Fig 2b). We attribute this to Pavlovian misbehaviour caused by the prospect of a large reward, consistent with the faster ultimate reaction time on successful large-reward trials in both Go and No-Go conditions (Fig 2c). Mirroring the case of active avoidance [14], on successful NGL trials, after a minor peak, there was a suppression of DA below baseline during the No-Go period (followed by a rise at movement initiation), despite the prospect of large reward (Fig 2d); by contrast, on successful GL trials, there was a marked early increase.

Again, we write *s*_pre_ for the state before the disambiguating cue, with *V*(*s*_pre_) ≃ 0, and *s*_*χ*_, *χ* ∈ {gs, gl, ngs, ngl}, for the states inspired by the respective cues. Partial success on NGL trials, and thus large rewards, would make *V*(*s*_ngl_) > 0, with

$$\begin{array}{c} \delta_{\text{ngl}}^{}=V\left(s_{\text{ngl}}\right)-V\left(s_{\text{pre}}\right)>0, \end{array}$$ (4)

promoting Pavlovian action, No-Go failure, and ultimately a decrease in *V*(*s*_ngl_)—lessening the misbehaviour. This slow negative feedback could even lead to oscillations.

In this case, we consider cognitive control as instilling a counterfactual state *s*_succ_ with *V*(*s*_succ_) ≫ 0 quantifying the full value of succeeding in the No-Go requirement. Then

$$\begin{array}{c} \delta_{\text{ngl}}^{}=V\left(s_{\text{ngl}}\right)-V\left(s_{\text{succ}}\right)<0, \end{array}$$ (5)

again harmonizing Pavlovian and instrumental control, this time by facilitating *inaction*. This amounts to moving the origin of the affective circumplex to the positive value associated with presumed NGL success (Fig 2e), switching the sign of the TD error (Fig 2f) and leading to suppression of DA release. The brief initial increase on successful NGL trials (Fig 2g) might arise before control is exerted, something we would need a finer timescale model to examine. Here, control failure, associated with failed NGL trials, should lead to enhanced DA release (Fig 2g, inset). Apparent trends in this direction were not, however, found to be significant (S1 Fig), though the relatively small sample size and large variability in the voltammetry signal may obscure such differences.

### Stability of reframing

An important remaining problem with the proposed reframing is the apparent absence of learning. For instance, if the DA signal of Eq (3) is positive, why does normal plasticity, associated with conventional TD learning, not zero out this egregious prediction error?

One possibility is that downstream systems might be informed directly about the counterfactual status of the reframing, and so avoid untoward plasticity. One could only speculate as to how this information could flow and take effect.

A second possibility is that cortico-striatal plasticity is confined to precise temporal windows, occasioned for instance by the activity of tonically active cholinergic neurons [43–46]. This window could be explicitly closed as part of the operation of control and so avoid the undesired plasticity.

Third, there might instead be an active mechanism associated with opponency [18, 20, 33](see also [12, 34]). That is, rather than

$$\begin{array}{c} \Delta V(s_{\text{fail}})\propto \delta_{\text{shk}}^{}, \end{array}$$ (6)

which would adjust *V*(*s*_fail_) towards 0 until *δ*_shk_ = 0, cancelling out the reframing mechanism, we would have

$$\Delta V\left(s_{\text{fail}}\right)\propto \delta_{\text{shk}}^{}-{\tilde{\delta }}_{\text{shk}}^{},$$ (7)

for an opponent prediction error ${\tilde{\delta }}_{\text{shk}}$. Then, Δ*V*(*s*_fail_) = 0 when

$$\delta_{\text{shk}}={\tilde{\delta }}_{\text{shk}},$$ (8)

rendering reframing stable. The same argument can be made for *s*_succ_ in the No-Go case.

This last perspective elucidates other cases with apparently non-zero asymptotic DA. Thus, the evidence from tasks demanding substantial work from subjects is that DA release does not inversely covary (at least strongly) with demands on vigour [47], but that compromising DA (e.g., by selective lesions [11]) compromises the willingness of subjects to overcome substantial effort costs in their active responding. If we imagine that those effort costs are conveyed by opponent terms such as ${\tilde{\delta }}_{\text{shk}}$, then the net influence on action in the striatum would depend on $\delta_{\text{shk}}-{\tilde{\delta }}_{\text{shk}}$, which would evidently be compromised by DA deficits.

The opponent might also help resolve a tension in our model of active avoidance between the apparent consistency of good-avoiders’ behaviour with negligible Pavlovian influence (i.e., *ω* ≈ 0) and the putative origin of positive DA on shock trials in the deployment of control. Instead of having no effect, as at present in the model, control might also be necessary for the good avoiders to overcome any possible Pavlovian misbehaviour arising from the opponent. Of course, control might also influence the opponent [48], making for a rich palette of possible interactions.

Key areas for future work include modelling the cost [49], learning [50], and anatomical realization (putatively involving the anterior cingulate cortex [51]) of cognitive control, along with the likely (and recursive) mesocortical DA influence over its prefrontal operation [52, 53]; addressing the ultimate habitization, at least for the avoidance case, of the relevant action and obviation of cognitive control [54]; encompassing the known spatial [55, 56] and temporal [57] heterogeneity in DA release; capturing the fuller temporal extent of the DA signal rather than just the cue-associated response, including its persistence until the time of reward (cf. Fig 1d); explaining the effects of pharmacological manipulation in the Go/No-Go task [58, 59]; and incorporating the dorsal striatum, with its suggested focus on the instrumental components of control, and its own dopaminergically-impacted bias in favour of action [12]. Even more generally, the counterfactual states that are instantiated through the medium of cognitive control could have implications beyond DA—for instance engaging default, Pavlovian, policies that are more specific than just activation or inhibition.

In sum, we have suggested a neurocomputational architecture in which simple rules coupling action and valence are subject to a form of cognitive control whose mode of action exploits this very coupling.

## Methods

### General model

**Fig 3:**
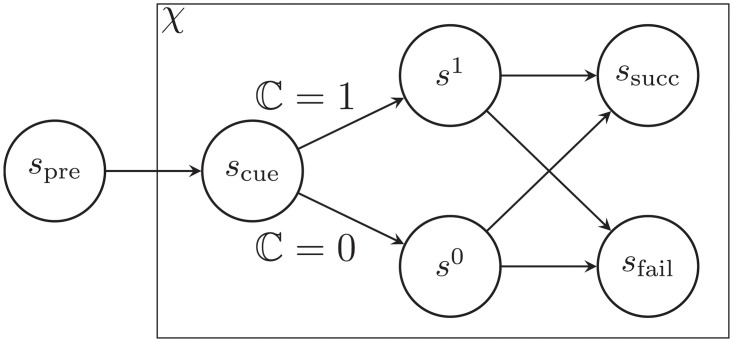
General model. See description in text.

Both tasks are modelled in essentially the same way (Fig 3). First, an ‘internal’ decision is made when a cue arrives, at state *s*_cue_, about whether to apply self-control (C=1) or not (C=0). There then follows an ‘external’ decision about the physical action at state *s*^1^ or *s*^0^, as appropriate. It is ultimately the physical action that determines success (to *s*_succ_) or failure (to *s*_fail_) for the current trial. Following the inter-trial interval (ITI), and any additional time penalty for failure, the next trial begins.

The recurrent nature of the tasks, and the fact that faster responses on the current trial can generally increase the rate of rewards (or punishments) by hastening opportunities to earn future outcomes in subsequent trials, mean that it is natural to employ an average-reward framework for the analysis. As shown in previous work (e.g., [19, 60]), the average reward rate *ρ* then becomes an important factor in promoting vigour by effectively determining the opportunity cost of time.

We denote trial type by *χ*. For simplicity, we assume that the initial, internal decision is made only on the trial types of most interest, i.e., on shock trials (*χ* = shk) in the mixed-valence task, and on No-Go large-reward (NGL) trials (*χ* = ngl) in the Go/No-Go task. Thus, we assume that the default choice is always C=0, only possibly deviating on these particular trial types.

The choice of whether to apply self-control or not is modelled in a very simple way. For shock and NGL trials, we simply assume that there is a fixed probability *κ*_*χ*_ of applying self-control,

$$\begin{array}{c} P_{\chi }\left(C=1\right)=\kappa_{\chi }, \end{array}$$ (9)

with this probability being fit to summary measures of the data as described below (‘Model-fitting’).

As described in the main text, the importance of this internal choice is its effect on the cue-elicited temporal difference (TD) error. For C=0, we assume that *s*^0^ is in essence an extension of the cue state, and so there is really no change to the TD error elicited by cue onset, i.e.,

$$\begin{array}{c} \delta_{\chi }^{}\left(s^{0}\right)\equiv V_{\chi }\left(s_{\text{cue}}\right)-V\left(s_{\text{pre}}\right). \end{array}$$ (10)

By contrast, for C=1, we assume that this is transformed to

$$\begin{array}{c} \delta_{\chi }\left(s^{1}\right)\equiv V_{\chi }\left(s_{\text{cue}}\right)-b_{\chi }, \end{array}$$ (11)

where *b*_*χ*_ is the trial-specific baseline/control signal that implements the putative reframing. As argued in the main text, we assume that *b*_*χ*_ is precisely the disutility of the shock that will be experienced on a shock trial if the animal fails to press, or is the utility of the large reward that stands to be lost on a NGL trial if the animal fails to maintain fixation.

In describing the models in greater detail below, we make use of the following common notation:

- *R*_*χ*_(*s*): immediate expected utility in current state *s* and trial type *χ*.
- *T*_*χ*_(*s*)/*T*_*χ*_(*s*, *a*, *τ*): expected time until the next state from current state *s* and trial type *χ*, potentially also conditioned on action (*a*, *τ*).

### Mixed-valence task

In this case [14], the trial types are *χ* ∈ {rew, neu, shk}, and we assume that the animal’s external choice is between *press* and *other*, where *other* can be thought of as some alternative activity that the animal may choose to engage in (e.g., grooming) and which may itself be rewarding, but will mean that the animal fails to press on the current trial.

In the experiment of [14], there was a 5 s interval between cue onset and the insertion of the response lever; in the model, for simplicity, we assume that the external choice is made at the time of the cue, and that implementation of that choice only begins at lever insertion. Following our previous work [20], we assume that successfully pressing on a reward trial delivers positive utility *r*_rew_ = 4, while failing to press on a shock trial leads to delivery of a punishing shock with disutility *r*_shk_ = −10. Briefly (noting that we do not seek to capture the DA concentration quantitatively), the unpleasantness of the shock is assumed to be greater in magnitude than the pleasantness of the reward based on the (dopaminergic) evidence that neutral trials had relatively positive value for poor avoiders, in spite of an average rate of successful avoidance of around 50%; roughly speaking, for this to hold under the model, the magnitude of the disutility of shock would need to be more than twice the utility of the reward, so that the average reward rate is negative and (therefore) the differential value associated with neutral trials is positive (see [20] for more detail).

If the animal chooses *press*, we assume it also chooses a latency *τ*_min_ ≤ *τ* ≤ *τ*_max_ for press completion, with *τ*_min_ = 0.5 s (for rough consistency with the data, although animals can certainly act more swiftly) and *τ*_max_ = 20 s. The latter may seem implausibly long, but also means we do not exclude the possibility that the animal chose to *press* but did not manage to complete it (e.g., due to freezing) before the *τ*_*D*_ = 10 s deadline. The (differential) value of pressing with latency *τ* is then

$$\begin{array}{c} Q_{\chi }\left(s,press,\tau \right)=\frac{c_{p}}{\tau }+V_{\chi }\left(s^{\prime }\right)-\rho T_{\chi }\left(s,press,\tau \right), \end{array}$$ (12)

where *c*_*p*_ ≤ 0 is the assumed (hyperbolic) vigour cost associated with choosing to press at latency *τ* (cf. [60]); *ρ* is the average reward rate; *s* ∈ {*s*_0_, *s*_1_} (noting that the instrumental values are the same for these states);

$$s^{\prime }=\{\begin{array}{cc} s_{\text{succ}} & \text{if}\;\tau \leq \tau_{D},\\ s_{\text{fail}} & \text{otherwise;} \end{array}$$

and

$$T_{\chi }\left(s,press,\tau \right)=\{\begin{array}{cc} \tau & \text{if}\;\tau \leq \tau_{D},\\ \tau_{D} & \text{otherwise.} \end{array}$$

For *other*, we simply assume, again for *s* ∈ {*s*_0_, *s*_1_}, that

$$\begin{array}{c} Q_{\chi }\left(s,other\right)=r_{O}\tau_{D}+V_{\chi }\left(s_{\text{fail}}\right)-\rho \tau_{D}, \end{array}$$ (13)

for some fixed utility rate *r*_*O*_ ≥ 0.

We assume that both instrumental and Pavlovian factors can affect speed of pressing. For presentational purposes in the main text, we refer to a single parameter *ω* that mediates the Pavlovian influence. However, in the model, we introduce a set of weights {*w*}, all *w* ≥ 0, that allow instrumental and Pavlovian factors to be balanced, but permitting Pavlovian influences on decisions about action vs. latency, and for positive vs. negative TD errors to differ (cf. [20]). This does not alter the central argument, and is a simple stand-in for the complexities of how these influences are mediated. In particular, we assume the distribution of pressing latencies to follow

$$\begin{array}{c} p_{\chi }\left(\tau \right)\propto \text{exp}\{w^{\tau_{i}}Q_{\chi }\left(s,press,\tau \right)-(w^{\tau_{v}^{+}}{\left[\delta_{\chi }\left(s\right)\right]}_{+}\tau +w^{\tau_{v}^{-}}{\left[\delta_{\chi }\left(s\right)\right]}_{-}\tau )\}, \end{array}$$ (14)

where $w^{\tau_{i}}$ is an instrumental weight, and $\left(w^{\tau_{v}^{+}},w^{\tau_{v}^{-}}\right)$ are Pavlovian weights that modulate the effect of positive and negative TD errors, respectively. This means that a positive TD error [*δ*_*χ*_(*s*)]_+_ will tend to speed up responding (since the value of a shorter *τ* will be penalized less by the term $-w^{\tau_{v}^{+}}{\left[\delta_{\chi }\left(s\right)\right]}_{+}\tau$, which is negative), while a negative TD error [*δ*_*χ*_(*s*)]_−_ will tend to slow responding down (since the value of a longer *τ* will be boosted more by the term $-w^{\tau_{v}^{-}}{\left[\delta_{\chi }\left(s\right)\right]}_{-}\tau$, which is positive).

The overall instrumental value of pressing is then assumed to be

$$\begin{array}{c} Q_{\chi }\left(s,press\right)=\int_{\tau_{\text{min}}}^{\tau_{\text{max}}}p_{\chi }\left(\tau \right)Q_{\chi }\left(s,press,\tau \right)\;d\tau , \end{array}$$ (15)

and the choice between *press* and *other* similarly assumed to be influenced by both instrumental and Pavlovian factors,

$$\begin{array}{c} P_{\chi }\left(press\right)\propto \text{exp}\{w^{i}\Delta Q_{\chi }\left(s\right)+w^{p+}{\left[\delta_{\chi }\left(s\right)\right]}_{+}+w^{p-}{\left[\delta_{\chi }\left(s\right)\right]}_{-}\}, \end{array}$$ (16)

where, again, *w*^*i*^ is an instrumental weight and (*w*^*p*+^, *w*^*p*−^) are Pavlovian weights for positive and negative TD errors; and Δ*Q*_*χ*_(*s*) ≡ *Q*_*χ*_(*s*, *press*) − *Q*_*χ*_(*s*, *other*). Thus, a positive TD error will tend to increase task engagement by boosting the probability of pressing, while a negative TD error will tend to decrease task engagement by instead boosting the probability of choosing *other*.

Note that there are two possible reasons for not pressing in the model. One is choosing *press* but failing to complete the press in time, for example because of a tendency to freeze (which we model implicitly via Eq 14), while the other is by choosing *other*. The latter choice could be purely instrumental (i.e., the reward and effort associated with the lever press, including savings on opportunity cost of time, is not sufficiently better than the alternative) or also involve Pavlovian factors (e.g., it makes instrumental sense to press the lever, but a negative TD error promotes disengagement via a form of disappointment or frustration—via Eq 16).

The values of success and failure states are respectively

$$\begin{array}{c} V_{\chi }\left(s_{\text{succ}}\right)=R_{\chi }\left(s_{\text{succ}}\right)-\rho T_{\chi }\left(s_{\text{succ}}\right), \end{array}$$ (17)

$$\begin{array}{c} V_{\chi }\left(s_{\text{fail}}\right)=R_{\chi }\left(s_{\text{fail}}\right)-\rho T_{\chi }\left(s_{\text{fail}}\right), \end{array}$$ (18)

where *R*_rew_(*s*_succ_) = *r*_rew_ = 4 for a successful reward trial, and zero otherwise; *R*_shk_(*s*_fail_) = *r*_shk_ = −10 for a failed shock trial, and zero otherwise; and *T*_*χ*_(*s*_succ_) = *T*_*χ*_(*s*_fail_) = 20 s, ∀*χ*, is the ITI.

### Go/No-Go task

In this case [15], the task demands a slightly different choice structure. As in the mixed-valence case, there is an initial internal choice about self-control. However, we then assume that the next immediate choice facing the animal is *when to leave the nose-poke*. That is, the animal simply chooses a time *τ* to leave the nose-poke. The trial types are *χ* ∈ {gs, gl, ngs, ngl}; the value of leaving at time *τ* for both *s* ∈ {*s*^0^, *s*^1^} is assumed to be

$$\begin{array}{c} Q_{\chi }\left(s,leave,\tau \right)=\{\begin{array}{cc} c_{f}\tau +\frac{c_{l}}{\tau }+V_{\chi }^{\text{out}}\left(\tau \right)-\rho \tau , & \chi \in \{\text{gs},\text{gl}\}\\ c_{f}\tau +\frac{c_{l}}{\tau }+P\left(succ|\tau \right)V_{\chi }\left(s_{\text{succ}}\right)\\ \;\;\;\;\;+\left[1-P\left(succ|\tau \right)\right]V_{\chi }\left(s_{\text{fail}}\right)-\rho \tau , & \chi \in \{\text{ngs},\text{ngl}\}, \end{array} \end{array}$$ (19)

where *c*_*f*_ ≤ 0 is a cost rate assumed to be associated with maintaining fixation in the nose-poke (e.g., reflecting a decreased ability to monitor for danger); *c*_*l*_ ≤ 0 is a cost rate associated with the vigour of leaving (i.e., shorter latencies are assumed more energetically demanding); $V_{\chi }^{\text{out}}\left(\tau \right)$ is the value on Go trials of having exited the nose-poke at time *τ*; and P(succ|τ)=U(1.7s,1.9s) is the probability of success on No-Go trials of exiting the nose-poke at time *τ* (i.e., the probability that the tone has turned off before exiting—see [15]). We should note here that even if the animal chooses to leave at particular time *τ* and the tone turns off *before* this time, we assume the animal will still in fact exit at time *τ* and incur the full costs $c_{f}\tau +\frac{c_{l}}{\tau }-\rho \tau$. In other words, we assume that the unfolding of the animal’s leaving is non-interruptible. If we assumed that the animal could be interrupted by the tone’s turning off and pay only a fractional cost of what it actually chose (cf. [19]), then it could make sense here to simply choose the slowest possible leaving time (which would have the lowest expected cost and, if implemented, would never result in leaving too early). Under the current formulation, the animal needs to balance, on the one hand, the vigour cost and possibility of failure if it leaves quickly and, on the other, the fixation cost and opportunity cost of time if it leaves slowly.

Just as with pressing latencies in the mixed-valence case (cf. Eq 14), we assume that the distribution of leaving times is influenced by both instrumental and Pavlovian factors,

$$\begin{array}{c} p_{\chi }\left(\tau |s,leave\right)\propto \text{exp}\{w^{\tau_{i}}Q_{\chi }\left(s,leave,\tau \right)-(w^{\tau_{v}^{+}}{\left[\delta_{\chi }\left(s\right)\right]}_{+}\tau +w^{\tau_{v}^{-}}{\left[\delta_{\chi }\left(s\right)\right]}_{-}\tau )\}, \end{array}$$ (20)

so that a positive TD error promotes leaving earlier, while a negative TD error promotes leaving later.

While the choice of leaving latency determines success or failure on No-Go trials (success is signalled by reward delivery, and failure by the turning on of the houselight—see [15]), we assume that on Go trials, an additional choice is required. That is, on exiting the nose-poke, the animal additionally chooses—as in the mixed-valence task—whether to subsequently *press* the lever or perform some *other* activity. Again, we can consider the value of pressing at different latencies, though this now depends on the time at which the animal exited the nose-poke; letting *t* denote the time that has elapsed since cue onset, the value of choosing to press at latency *τ* at that time (for *χ* ∈ {gs, gl}) is assumed to be

$$\begin{array}{c} Q_{\chi }\left(t,press,\tau \right)=\{\begin{array}{cc} \frac{c_{p}}{\tau }+V_{\chi }\left(s_{\text{succ}}\right)-\rho \tau , & \text{if}\;\tau \leq \tau_{D}-t;\\ \frac{c_{p}}{\tau }+V_{\chi }\left(s_{\text{fail}}\right)-\rho \left(\tau_{D}-t\right) & \text{otherwise,} \end{array} \end{array}$$ (21)

where, again, *c*_*p*_ ≤ 0 is the (vigour) cost rate associated with pressing. The instrumental value of choosing *other* is assumed to be

$$\begin{array}{c} Q_{\chi }\left(t,other\right)=\left(r_{O}-\rho \right)\left(\tau_{D}-t\right)+V_{\chi }\left(s_{\text{fail}}\right), \end{array}$$ (22)

since the duration of enjoyment of this alternative activity (at reward rate *r*_*O*_ ≥ 0), and accruing opportunity cost of time (at rate *ρ*), is *τ*_*D*_ − *t* seconds.

Assuming that choice of pressing latencies given choice of *press* is governed by a softmax function,

$$\begin{array}{c} p_{\chi }\left(\tau |t,press\right)\propto \text{exp}(\beta^{\tau }Q_{\chi }\left(t,press,\tau \right)), \end{array}$$ (23)

and

$$\begin{array}{c} Q_{\chi }\left(t,press\right)=\int_{\tau_{\text{min}}}^{\tau_{\text{max}}}p_{\chi }\left(\tau |t,press\right)Q_{\chi }\left(t,press,\tau \right)\;d\tau , \end{array}$$ (24)

we assume that the probability of choosing *press*, as opposed to *other*, at time *t* is given by the logistic/softmax function

$$\begin{array}{c} P_{t}\left(press\right)=\frac{1}{1+\text{exp}(-\beta (Q_{\chi }\left(t,press\right)-Q_{\chi }\left(t,other\right)))}. \end{array}$$ (25)

Note that we therefore assume for simplicity that the choice between *press* and *other* on exiting the nose-poke is purely instrumentally-governed. The value of having left the nose-poke at time *t* on a Go trial is then

$$\begin{array}{c} V_{\chi }^{\text{out}}\left(t\right)=P_{t}\left(press\right)Q_{\chi }\left(t,press\right)+\left[1-P_{t}\left(press\right)\right]Q_{\chi }\left(t,other\right). \end{array}$$ (26)

The value of success states are

$$\begin{array}{c} V_{\chi }\left(s_{\text{succ}}\right)=\{\begin{array}{cc} r_{S}-\rho \tau_{I}, & \text{for}\;\text{small-reward}\;\text{trials;}\\ r_{L}-\rho \tau_{I}, & \text{for}\;\text{large-reward}\;\text{trials,} \end{array} \end{array}$$ (27)

where *τ*_*I*_ = 5 s is the ITI. For fail states, we have for all trial types

$$\begin{array}{c} V_{\chi }\left(s_{\text{fail}}\right)=-\rho \left(\tau_{I}+\tau_{P}\right), \end{array}$$ (28)

where *τ*_*P*_ = 5 s is the penalty timeout.

### Dopamine

As described in the main text, we assume that the Pavlovian influence operates via the TD prediction error *δ*_*χ*_(*s*). While a rather large body of evidence supports the idea that this quantity is signalled by the activity of midbrain DA neurons (see citations in main text), there is also evidence that DA activity may not be its sole representational substrate. Indeed, one interesting feature of the results in [14] is the apparent absence of any immediate dip in DA in response to the shock cue (perhaps before control is deployed) on successful avoidance trials.

We emphasize that we seek to model only DA transients associated with the task cues that differentiate between trial types, and not the later release which is contemporaneous with movements and/or delivery of outcomes. We have therefore focused on particular epochs proximal to cue onset in each experiment: the first 5 s following cue onset, and prior to lever insertion, in the mixed-valence task; and the first 3 s following cue onset in the Go/No-Go task. We shaded off the unmodelled times in Figs 1 and 2. Accounting for the full trajectory of DA release over a trial would require a more complex model, possibly encompassing factors that we have considered elsewhere [61].

We follow [33] in assuming that DA indeed only signals part of the full TD error, and principally signals transitions that are better than expected. In particular, similar (but not identical) to [33], we assume that the dopaminergic component $\delta_{\chi }^{\text{DA}}$ is given by

$$\begin{array}{c} \delta_{\chi }^{\text{DA}}=\alpha [\delta_{\chi }^{}{]}_{+}+\left(1-\alpha \right)[\delta_{\chi }^{}{]}_{-}, \end{array}$$ (29)

with *α* = 0.8 throughout, for simplicity. In [33] and later work [18], it was explicitly suggested that punishers and their predictors would engage non-dopaminergic substrates, notably serotonin. However, current views of the landscape of interactions between dopamine and serotonin point to many complexities (see [62]), and this can at most be a part of the overall picture.

We additionally need to consider how the quantity $\delta_{\chi }^{\text{DA}}$, putatively represented by the firing of DA neurons, is reflected in changes in DA release measured in the accumbens (NAc). Here, we simply assume that this term is convolved with an alpha function (cf. [61]),

$$\begin{array}{c} f\left(t\right)=\frac{t}{\xi }e^{1-\frac{t}{\xi }}, \end{array}$$ (30)

with time constant *ξ* = 0.7 s, so that changes in DA concentration relative to baseline are given by

$$\begin{array}{c} \Delta \left[\text{DA}\right]\propto \delta_{\chi }^{\text{DA}}*f\equiv \int_{-\infty }^{\infty }\delta^{\text{DA}}\left(s\right)f\left(t-s\right)\;ds. \end{array}$$ (31)

To allow for the possibility that it may take some non-trivial amount of time for control/reframing to be applied (as possibly hinted by the results of Syed et al. [15] on successful NGL trials), for trials on which this is the case (i.e., C=1), we assume that we initially have $\delta_{\chi }^{\text{DA}}\left(s^{0}\right)$ at cue onset, but that this is followed by $\delta_{\chi }^{\text{DA}}\left(s^{1}\right)$ after a short, subsecond delay (we arbitrarily set this to *τ*_delay_ = 250 ms).

Note that to assess model-derived DA responses on success vs. failed trials separately, we compute the posterior probability of having employed control given success (since success and failure could occur whether or not control was employed):

$$\begin{array}{cc} P_{\chi }\left(C=1|\text{success}\right) & \propto P_{\chi }\left(\text{success}|C=1\right)P_{\chi }\left(C=1\right)\\ & =P_{\chi }\left(\text{success}|C=1\right)\kappa_{\chi }, \end{array}$$ (32)

and the probability of having employed control given failure,

$$\begin{array}{cc} P_{\chi }\left(C=1|\text{fail}\right) & \propto P_{\chi }\left(\text{fail}|C=1\right)P_{\chi }\left(C=1\right)\\ & =P_{\chi }\left(\text{fail}|C=1\right)\kappa_{\chi }. \end{array}$$ (33)

Thus, the average DA signal on successful trials is a mixture of those on which control was employed (with inferred probability $P_{\chi }\left(C=1|\text{success}\right)$) and those on which it was not (with probability $P_{\chi }\left(C=0|\text{success}\right)=1-P_{\chi }\left(C=1|\text{success}\right)$). Analogous reasoning allows for derivation of the average DA signal on failed trials.

### Model-fitting

For a given set of parameters **x**, the self-consistent set of differential state values and associated behaviour can be found using value iteration [63]. For each task, we fitted parameters (see Tables 1 and 2) to minimize the difference between animal and model behaviour. In particular, we defined an error function

$$\begin{array}{c} E\left(x\right)=\sum_{\chi }|e_{\chi }^{\text{succ}}|+w_{\text{rtc}}\sum_{\chi }\frac{|e_{\chi }^{\text{rtc}}|}{s_{\chi }^{\text{rtc}}}+w_{\text{rte}}\sum_{\chi }\frac{|e_{\chi }^{\text{rte}}|}{s_{\chi }^{\text{rte}}}+w_{\text{DA}}e^{\text{DA}}+w_{reg}\left\|x\right\|, \end{array}$$ (34)

where $e_{\chi }^{\text{succ}}$, $e_{\chi }^{\text{rtc}}$, and $e_{\chi }^{\text{rte}}$ are the differences between the average performance of the animals and the model (for expected success rates, reaction times for correct trials, and reaction times for error trials, respectively); $s_{\chi }^{\text{rtc}}$ and $s_{\chi }^{\text{rte}}$ are the standard errors from the experimental data (so that the larger the standard error, the less importance we place on precise fitting of that measurement); *w*_rtc_ ≥ 0 and *w*_rte_ ≥ 0 are weights that determine the relative importance we give to fitting the reaction times compared to the success rates (in practice, we set these to the same value: for the mixed-valence task, we fixed *w*_rtc_ = *w*_rte_ = 0.25; for Go/No-Go, we fixed *w*_rtc_ = *w*_rte_ = 0.018); and *w*_*reg*_ is a (L1) regularization weight that determines how strongly we wish to discourage large values in the parameters **x** (we set *w*_*reg*_ = 0.01 when fitting good avoidance in the mixed-valence task, since the instrumental weights can grow arbitrarily large in this case, and otherwise set it to zero). The term *e*^DA^ is an error term that we used only when fitting data from the mixed-valence task, and is specifically the absolute difference between peak DA for food and shock trials produced by the model—this was to better reproduce the striking similarity between food- and shock-trial DA transients observed in the data (we set *w*_DA_ = 0.1 for both good and poor avoiders).

**Table 1:** Model parameters used for mixed-valence task and best-fitting free parameter values for good- and poor-avoiders. {*r*_rew_, *r*_neu_, *r*_shk_}: utilities of outcomes on press trials for each trial type. *r*_*O*_: utility rate associated with action *other*. *c*_*p*_: vigour cost of pressing. *b*_shk_: baseline/control signal optionally applied on shock trials. *α*: mixture weight determining relationship between full TD error and its dopaminergic component. *τ*_delay_: putative delay associated with application of control/reframing. {*w*^*i*^, $w^{\tau_{i}}$}: instrumental weights. {*w*^*p*+^, *w*^*p*−^, $w^{\tau_{v}^{+}}$, $w^{\tau_{v}^{-}}$}: Pavlovian weights. *κ*: probability of deploying control/reframing.

model parameters			
fixed	free	good	poor
*r*_rew_ = 4	*w* ^*i*^	2.45	0.01
*r*_neu_ = 0	*w* ^*p*+^	0.00	0.82
*r*_shk_ = −10	*w* ^*p*−^	0.01	0.74
*r_O_* = 0	$w^{\tau_{i}}$	41.28	0.00
*c_p_* = −0.1	$w^{\tau_{v}^{+}}$	0.97	1.06
*b*_shk_ = *r*_shk_	$w^{\tau_{v}^{-}}$	0.00	0.00
*α* = 0.8	*κ*	0.36	0.50
*τ*_delay_ = 250 ms			

**Table 2:** Model parameters used for Go/No-Go task and best-fitting free parameter values. {*r*_*S*_, *r*_*L*_}: utilities of small and large rewards. *b*_ngl_: baseline/control signal optionally applied on NGL trials. *α*: mixture weight determining relationship between full TD error and its dopaminergic component. *τ*_delay_: putative delay associated with application of control/reframing. $w^{\tau_{i}}$: instrumental weight. $\left\{w^{\tau_{v}^{+}},w^{\tau_{v}^{-}}\right\}$: Pavlovian weights. {*β*^*τ*^, *β*}: inverse temperatures of softmax functions. *κ*: probability of deploying control/reframing. *r*_*O*_: utility rate associated with action *other*. {*c*_*f*_, *c*_*l*_, *c*_*p*_}: costs respectively associated with maintaining fixation, leaving, and pressing.

model parameters		
fixed	free	
*r_S_* = 1	$w^{\tau_{i}}$	1.94
*r_L_* = 2	$w^{\tau_{v}^{+}}$	11.63
*b*_ngl_ = *r_L_*	$w^{\tau_{v}^{-}}$	0.01
*α* = 0.8	*β* ^*τ*^	0.66
*τ*_delay_ = 250 ms	*β*	2.95
	*κ*	0.50
	*r*_*O*_	0.12
	*c*_*f*_	-0.44
	*c*_*l*_	-0.10
	*c*_*p*_	-0.00

This latter point deserves amplification. For the good avoiders, behaviour is consistent with a purely instrumental policy, with Pavlovian weights ≈ 0 (see Table 1). This implies that behaviour in the model does not vary with *δ*_*χ*_ (or therefore the value of *κ*)—the model reliably presses on shock trials in any case. *κ* is only identifiable when additionally considering how the model’s implied DA pattern compares with the data, hence the fitting weight *w*_DA_ above. Why though would control be at all necessary if behaviour were purely instrumental, since there would then apparently be no danger of Pavlovian misbehaviour? As mentioned in the main text, one possibility is that an opponent signal might also influence behaviour, which controlled dopamine release would be required to overcome. Indeed, note that good-avoiders still freeze in response to shock cues (cf. Figure 4i of [14]). We leave these subtleties to future work.

### Data analysis

The data from [15] were downloaded from https://data.mrc.ox.ac.uk. Following [15], data were smoothed using a 0.5 s moving window and baselined by subtracting the average signal during the 0.5 s period before cue onset. As a basic test of the hypothesis that the cue-evoked DA response would be greater on failed No-Go large-reward (NGL) trials than on successful NGL trials, we integrated the DA signal over the 1 s period immediately following cue-onset, averaged this measure for each session, and applied a (paired, one-tailed) *t*-test to these session-wise averages.

## Supporting information

S1 Fig DA release on successful vs. failed No-Go large-reward (NGL) trials. Average cue-aligned (at 0 s) change in DA release (±SEM) on successful vs. failed No-Go large-reward (NGL) trials in [15]; triangles indicate average RT for success (filled) vs. failure (unfilled) trials. Inset: average integrated DA signal (±SEM) over the first 1 s following cue-onset on successful (s) vs. failed (f) trials; *t*(8) = 1.04, *p* = .16 (n.s.), one-tailed.
(EPS)

## References

[1] Mackintosh NJ. Conditioning and associative learning. Oxford University Press; 1983.

[2] Dayan P, Niv Y, Seymour B, Daw ND. The misbehavior of value and the discipline of the will. Neural Networks. 2006;19(8):1153–60. doi: 10.1016/j.neunet.2006.03.002 16938432

[3] Bach DR, Dayan P. Algorithms for survival: A comparative perspective on emotions. Nature Reviews Neuroscience. 2017;. doi: 10.1038/nrn.2017.35 28360419

[4] Breland K, Breland M. The misbehavior of organisms. American Psychologist. 1961;16:681–684. doi: 10.1037/h0040090

[5] Hershberger WA. An approach through the looking-glass. Animal Learning and Behavior. 1986;14:443–451. doi: 10.3758/BF03200092

[6] Williams DR, Williams H. Auto-maintenance in the pigeon: sustained pecking despite contingent non-reinforcement. Journal of the experimental analysis of behavior. 1969;12(4):511–520. doi: 10.1901/jeab.1969.12-511 16811370 PMC1338642

[7] Crockett M, Clark L, Robbins TW. Reconciling the role of serotonin in behavioral inhibition and aversion: Acute tryptophan depletion abolishes punishment-induced inhibition in humans. Journal of Neuroscience. 2009;29(38):11993–11999. doi: 10.1523/JNEUROSCI.2513-09.2009 19776285 PMC2775933

[8] Guitart-Masip M, Beierholm UR, Dolan R, Duzel E, Dayan P. Vigor in the face of fluctuating rates of reward: an experimental examination. Journal of Cognitive Neuroscience. 2011;23(12):3933–3938. doi: 10.1162/jocn_a_00090 21736459

[9] Swart JC, Froböse MI, Cook JL, Geurts DEM, Frank MJ, Cools R, et al. Catecholaminergic challenge uncovers distinct Pavlovian and instrumental mechanisms of motivated (in)action. eLife. 2017;6(e22169). doi: 10.7554/eLife.22169 28504638 PMC5432212

[10] Schultz W, Dayan P, Montague PR. A neural substrate of prediction and reward. Science. 1997;275:1593–1599. doi: 10.1126/science.275.5306.1593 9054347

[11] Salamone JD, Correa M. The mysterious motivational functions of mesolimbic dopamine. Neuron. 2012;76:470–485. doi: 10.1016/j.neuron.2012.10.021 23141060 PMC4450094

[12] Collins AGE, Frank MJ. Opponent Actor Learning (OpAL): modeling interactive effects of striatal dopamine on reinforcement learning and choice incentive. Psychological Review. 2014;121(3):337–366. doi: 10.1037/a0037015 25090423

[13] Oleson EB, Cheer JF. On the role of subsecond dopamine release in conditioned avoidance. Frontiers in Neuroscience. 2013;7:101–109. doi: 10.3389/fnins.2013.00096 23759871 PMC3675318

[14] Gentry RN, Lee B, Roesch MR. Phasic dopamine release in the rat nucleus accumbens predicts approach and avoidance performance. Nature Communications. 2016;7(131154). doi: 10.1038/ncomms13154 27786172 PMC5095290

[15] Syed ECJ, Grima LL, Magill PJ, Bogacz R, Brown P, Walton ME. Action initiation shapes mesolimbic dopamine encoding of future rewards. Nature Neuroscience. 2016;19(1):34–36. doi: 10.1038/nn.4187 26642087 PMC4697363

[16] Mowrer OH. On the dual nature of learning: A reinterpretation of “conditioning” and “problem-solving”. Harvard Educational Review. 1947;17:102–150.

[17] Mowrer OH. Two-factor learning theory reconsidered, with special reference to secondary reinforcement and the concept of habit. Psychological Review. 1956;63(2):114–128. doi: 10.1037/h0040613 13310707

[18] Boureau YL, Dayan P. Opponency revisited: Competition and cooperation between dopamine and serotonin. Neuropsychopharmacology. 2011;36(1):74–97. doi: 10.1038/npp.2010.151 20881948 PMC3055522

[19] Dayan P. Instrumental vigour in punishment and reward. European Journal of Neuroscience. 2012;35(7):1152–1168. doi: 10.1111/j.1460-9568.2012.08026.x 22487044

[20] Lloyd K, Dayan P. Pavlovian-instrumental interactions in active avoidance: The bark of neutral trials. Brain research. 2019;1713:52–61. doi: 10.1016/j.brainres.2018.10.011 30308188

[21] Gentry RN, Lee B, Roesch MR. Phasic dopamine release in the rat nucleus accumbens predicts approach and avoidance performance. Nature Communications. 2016;7:131154. doi: 10.1038/ncomms13154 27786172 PMC5095290

[22] Dickinson A, Balleine B. Actions and responses: The dual psychology of behaviour. In: Eilan N, McCarthy RA, Brewer B, editors. Spatial representation: Problems in philosophy and psychology. Oxford: Blackwell; 1993. p. 277–293.

[23] Crockett MJ, Clark L, Robbins TW. Reconciling the role of serotonin in behavioral inhibition and aversion: acute tryptophan depletion abolishes punishment-induced inhibition in humans. Journal of Neuroscience. 2009;29(38):11993–11999. doi: 10.1523/JNEUROSCI.2513-09.2009 19776285 PMC2775933

[24] Guitart-Masip M, Duzel E, Dolan R, Dayan P. Action versus valence in decision making. Trends in Cognitive Sciences. 2014;18(4):194–202. doi: 10.1016/j.tics.2014.01.003 24581556 PMC3989998

[25] Huys QJM, Cools R, Gölzer M, Friedel E, Heinz A, Dolan RJ, et al. Disentangling the roles of approach, activation and valence in instrumental and pavlovian responding. PLoS Computational Biology. 2011;7(4):e1002028. doi: 10.1371/journal.pcbi.1002028 21556131 PMC3080848

[26] Watkins CJCH. Learning from Delayed Rewards. PhD Thesis, University of Cambridge. 1989;.

[27] Sutton RS. Learning to predict by the methods of temporal differences. Machine Learning. 1988;3(1):9–44. doi: 10.1007/BF00115009

[28] Montague PR, Dayan P, Sejnowski TJ. A framework for mesencephalic dopamine systems based on predictive hebbian learning. The Journal of Neuroscience. 1996;16(5):1936–1947. doi: 10.1523/JNEUROSCI.16-05-01936.1996 8774460 PMC6578666

[29] Starkweather CK, Uchida N. Dopamine signals as temporal difference errors: recent advances. Current Opinion in Neurobiology. 2021;67:95–105. doi: 10.1016/j.conb.2020.08.014 33186815 PMC8107188

[30] Kim HR, Malik AN, Mikhael JG, Bech P, Tsutsui-Kimura I, Sun F, et al. A unified framework for dopamine signals across timescales. Cell. 2020;183(6):1600–1616. doi: 10.1016/j.cell.2020.11.013 33248024 PMC7736562

[31] Berridge KC. The debate over dopamine’s role in reward: the case for incentive salience. Psychopharmacology. 2007;191:391–431. doi: 10.1007/s00213-006-0578-x 17072591

[32] McClure SM, Daw ND, Montague PR. A computational substrate for incentive salience. Trends in Neurosciences. 2003;26(8):423–428. doi: 10.1016/S0166-2236(03)00177-2 12900173

[33] Daw ND, Kakade S, Dayan P. Opponent interactions between serotonin and dopamine. Neural Networks. 2002;15:603–616. doi: 10.1016/S0893-6080(02)00052-7 12371515

[34] Jaskir A, Frank MJ. On the normative advantages of dopamine and striatal opponency for learning and choice. Elife. 2023;12:e85107. doi: 10.7554/eLife.85107 36946371 PMC10198727

[35] Bennett D, Davidson G, Niv Y. A model of mood as integrated advantage. Psychological Review. 2022;129(3):513–541. doi: 10.1037/rev0000294 34516150 PMC8917968

[36] Oleson EB, Gentry RN, Chioma VC, Cheer JF. Subsecond dopamine release in the nucleus accumbens predicts conditioned punishment and its successful avoidance. The Journal of Neuroscience. 2012;32(42):14804–14808. doi: 10.1523/JNEUROSCI.3087-12.2012 23077064 PMC3498047

[37] Wenzel JM, Oleson EB, Gove WN, Cole AB, Gyawali U, Dantrassy HM, et al. Phasic dopaminergic signals in the nucleus accumbens that cause active avoidance require endocannibinoid mobilization in the midbrain. Current Biology. 2018;28(9):1392–1404. doi: 10.1016/j.cub.2018.03.037 29681476 PMC5940536

[38] Kakade S, Dayan P. Dopamine: generalization and bonuses. Neural Networks. 2002;15:549–559. doi: 10.1016/S0893-6080(02)00048-5 12371511

[39] Mirenowicz J, Schultz W. Preferential activation of midbrain dopamine neurons by appetitive rather than aversive stimuli. Nature. 1996;379:449–451. doi: 10.1038/379449a0 8559249

[40] Day JJ, Roitman MF, Wightman RM, Carelli R. Associative learning mediates dynamic shifts in dopamine signaling in the nucleus accumbens. Nature Neuroscience. 2007;10(8):1020–1028. doi: 10.1038/nn1923 17603481

[41] Flagel SB, Clark JJ, Robinson TE, Mayo L, Czuj A, Willuhn I, et al. A selective role for dopamine in stimulus-reward learning. Nature. 2011;469(7328):53–57. doi: 10.1038/nature09588 21150898 PMC3058375

[42] Hart A, Clark JJ, Phillips PEM. Dynamic shaping of dopamine signals during probabilistic Pavlovian conditioning. Neurobiology of Learning and Memory. 2015;117:84–92. doi: 10.1016/j.nlm.2014.07.010 25172480 PMC4293327

[43] Lerner TN, Kreitzer AC. Neuromodulatory control of striatal plasticity and behavior. Current Opinion in Neurobiology. 2011;21(2):322–327. doi: 10.1016/j.conb.2011.01.005 21333525 PMC3092792

[44] Bradfield LA, Bertran-Gonzalez J, Chieng B, Balleine BW. The thalamostriatal pathway and cholinergic control of goal-directed action: Interlacing new with existing learning in the striatum. Neuron. 2013;79(1):153–166. doi: 10.1016/j.neuron.2013.04.039 23770257 PMC3863609

[45] Deffains M, Bergman H. Striatal cholinergic interneurons and cortico-striatal synaptic plasticity in health and disease. Movement Disorders. 2015;30(8):1014–1025. doi: 10.1002/mds.26300 26095280

[46] Franklin NT, Frank MJ. A cholinergic feedback circuit to regulate striatal population uncertainty and optimize reinforcement learning. Elife. 2015;4:e12029. doi: 10.7554/eLife.12029 26705698 PMC4764588

[47] Walton ME, Bouret S. What is the relationship between dopamine and effort? Trends in Neurosciences. 2019;42(2):79–91. doi: 10.1016/j.tins.2018.10.001 30391016 PMC6352317

[48] Maier SF, Watkins LR. Stressor controllability and learned helplessness: the roles of the dorsal raphe nucleus, serotonin, and corticotropin-releasing factor. Neuroscience & Biobehavioral Reviews. 2005;29(4-5):829–841. doi: 10.1016/j.neubiorev.2005.03.021 15893820

[49] Shenhav A, Botvinick MM, Cohen JD. The expected value of control: An integrative theory of anterior cingulate function. Neuron. 2013;79:217–240. doi: 10.1016/j.neuron.2013.07.007 23889930 PMC3767969

[50] Lieder F, Shenhav A, Musslick S, Griffiths TL. Rational metareasoning and the plasticity of cognitive control. PLoS Computational Biology. 2018;14(4):e1006043. doi: 10.1371/journal.pcbi.1006043 29694347 PMC5937797

[51] Carter CS, Botvinick MM, Cohen JD. The contribution of the anterior cingulate cortex to executive processes in cognition. Reviews in the Neurosciences. 1999;10(1):49–58. doi: 10.1515/REVNEURO.1999.10.1.49 10356991

[52] Yee DM, Braver TS. Interactions of motivation and cognitive control. Current Opinion in Behavioral Sciences. 2018;19:83–90. doi: 10.1016/j.cobeha.2017.11.009 30035206 PMC6051692

[53] Cools R. Chemistry of the adaptive mind: Lessions from dopamine. Neuron. 2019;104:113–131. doi: 10.1016/j.neuron.2019.09.035 31600509

[54] Cain CK. Avoidance problems reconsidered. Current Opinion in Behavioral Sciences. 2019;26:9–17. doi: 10.1016/j.cobeha.2018.09.002 30984805 PMC6456067

[55] de Jong JW, Fraser KM, Lammel S. Mesoaccumbal dopamine heterogeneity: What do dopamine firing and release have to do with it? Annual Review of Neuroscience. 2022;45:109–129. 10.1146/annurev-neuro-110920-011929 PMC9271543 35226827

[56] Menegas W, Akiti K, Amo R, Uchida N, Watabe-Uchida M. Dopamine neurons projecting to the posterior striatum reinforce avoidance of threatening stimuli. Nature Neuroscience. 2018;21(10):1421–1430. doi: 10.1038/s41593-018-0222-1 30177795 PMC6160326

[57] Mohebi A, Pettibone JR, Hamid AA, Wong JMT, Vinson LT, Patriarchi T, et al. Dissociable dopamine dynamics for learning and motivation. Nature. 2019;570(7759):65–70. doi: 10.1038/s41586-019-1235-y 31118513 PMC6555489

[58] Grima LL, Panayi MC, Härmson O, Syed ECJ, Manohar SG, Husain M, et al. Nucleus accumbens D1-receptors regulate and focus transitions to reward-seeking action. Neuropsychopharmacology. 2022;. doi: 10.1038/s41386-022-01312-6 PMC9283443 35478011

[59] Härmson O, Grima LL, Panayi MC, Husain M, Walton ME. 5-HT2C receptor perturbation has bidirectional influence over vigour and restraint. Psychopharmacology. 2022;239:123–140. doi: 10.1007/s00213-021-05992-8 34762147 PMC8770415

[60] Niv Y, Daw ND, Joel D, Dayan P. Tonic dopamine: opportunity costs and the control of response vigor. Psychopharmacology. 2007;191(3):507–520. doi: 10.1007/s00213-006-0502-4 17031711

[61] Lloyd K, Dayan P. Tamping Ramping: Algorithmic, Implementational, and Computational Explanations of Phasic Dopamine Signals in the Accumbens. PLoS Computational Biology. 2015;11(12):e1004622. doi: 10.1371/journal.pcbi.1004622 26699940 PMC4689534

[62] Peters KZ, Cheer JF, Tonini R. Modulating the neuromodulators: dopamine, serotonin and the endocannabinoid system. Trends in Neurosciences. 2021;44(6):464–477. doi: 10.1016/j.tins.2021.02.001 33674134 PMC8159866

[63] Mahadevan S. Average reward reinforcement learning: Foundations, algorithms, and empirical results. Machine Learning. 1996;22:159–196. doi: 10.1023/A:1018064306595

